# Dysfunction of Resting-State Functional Connectivity of Amygdala Subregions in Drug-Naïve Patients With Generalized Anxiety Disorder

**DOI:** 10.3389/fpsyt.2021.758978

**Published:** 2021-10-13

**Authors:** Mei Wang, Lingxiao Cao, Hailong Li, Hongqi Xiao, Yao Ma, Shiyu Liu, Hongru Zhu, Minlan Yuan, Changjian Qiu, Xiaoqi Huang

**Affiliations:** ^1^Mental Health Center and Psychiatric Laboratory, West China Hospital of Sichuan University, Chengdu, China; ^2^Huaxi MR Research Center (HMRRC), Functional and Molecular Imaging Key Laboratory of Sichuan Province, Department of Radiology, West China Hospital, Sichuan University, Chengdu, China; ^3^Psychoradiology Research Unit of the Chinese Academy of Medical Sciences, West China Hospital of Sichuan University, Chengdu, China

**Keywords:** generalized anxiety disorder, amygdala, resting-state functional connectivity, state anxiety, trait anxiety

## Abstract

**Objective:** Although previous studies have reported on disrupted amygdala subregional functional connectivity in generalized anxiety disorder (GAD), most of these studies were conducted in GAD patients with comorbidities or with drug treatment. Besides, whether/how the amygdala subregional functional networks were associated with state and trait anxiety is still largely unknown.

**Methods:** Resting-state functional connectivity of amygdala subregions, including basolateral amygdala (BLA) and centromedial amygdala (CMA) as seed, were mapped and compared between 37 drug-naïve, non-comorbidity GAD patients and 31 age- and sex-matched healthy controls (HCs). Relationships between amygdala subregional network dysfunctions and state/trait anxiety were examined using partial correlation analyses.

**Results:** Relative to HCs, GAD patients showed weaker functional connectivity of the left BLA with anterior cingulate/medial prefrontal cortices. Significantly increased functional connectivity of right BLA and CMA with superior temporal gyrus and insula were also identified in GAD patients. Furthermore, these functional connectivities showed correlations with state and trait anxiety scores.

**Conclusions:** These findings revealed abnormal functional coupling of amygdala subregions in GAD patients with regions involved in fear processing and emotion regulation, including anterior cingulate/medial prefrontal cortex and superior temporal gyrus, which provide the unique biological markers for GAD and facilitating the future accurate clinical diagnosis and target treatment.

## Introduction

Generalized anxiety disorder (GAD) is a common anxiety disorder characterized by excessive, pervasive, and uncontrollable anxiety, with a lifetime prevalence of about 5.7% (1). GAD is associated with substantial mental impairment affecting daily physical, psychological, and social functioning (2). The amygdala, as a core structure involved in fear processing and emotion regulation, is considered to be central to the pathophysiology of GAD (3). Although a lot of previous studies have reported functional abnormalities of amygdala in GAD (4–7), whether/how the intrinsic functional changes of amygdala in drug-naïve GAD are still not well-investigated, especially at the functionally heterogeneous subregional level.

The structural and functional heterogeneity of amygdala have been well-documented (8–10). The amygdala is commonly classified into the basolateral amygdala (BLA) and centromedial amygdala (CMA), which have different functions through distinct connectivity patterns (11). The BLA receives and integrates cortical input information from multiple brain systems for perception, evaluation, and memory formation of emotionally salient events. The CMA, in contrast, mainly receives modulatory inputs from the BLA and orbitofrontal cortex and projects to the brainstem, cerebellum, and hypothalamus for behavioral and physiological aspects of emotion processing and associative learning (11–14). These connectivity patterns of amygdala subregions have been proven by tractography, task-based and resting-state functional magnetic resonance imaging (9, 10, 13).

Resting-state functional connectivity (RSFC) analysis is a powerful method to examine intrinsic functional connectivity abnormalities in various mental disorders, which delineates the functional architecture of intrinsically coupled brain networks (15–20). By using RSFC analysis, disruptions of the amygdala subregional functional connectivity have been reported in patients with GAD. For example, BLA and CMA connectivity patterns were found to be significantly less distinct in adults with GAD (21). Roy et al. found that adolescents with GAD showed disrupted amygdala subregional functional networks that included regions in medial prefrontal cortex, insula, and cerebellum (22). However, these findings were concluded from a relatively small sample of GAD patients with comorbidities or with drug treatment, which need to be further validated.

Moreover, GAD patients are frequently involved in a state of anxiety, a measure of the intensity of anxiety experienced on comparatively short timescales. If state anxiety is deployed appropriately, it serves as an adaptive function priming individuals to detect and respond to danger. Compared with state anxiety, trait anxiety is derived from self-report questionnaires to measure the frequency of anxiety symptoms that are experienced by an individual or the general personality properties (23, 24). State and trait anxiety are different, and elevated levels of trait anxiety are a risk factor for the development of clinical anxiety disorders (25). Yet, the associations of amygdala subregional functional connectivity with state and trait anxiety have rarely been investigated in GAD. Thus, to depict the relationship of amygdala subregional functional connectivity patterns with state and trait anxiety in drug-naïve patients may better identify GAD neuropathological basis.

In the current study, we aimed to explore the specific amygdala subregional functional connectivity alterations in drug-naïve, non-comorbidity patients with GAD compared with age- and sex-matched healthy controls (HCs). Then, we evaluated the associations of amygdala subregional connectivity dysfunctions with different anxiety types. Based on the above mentioned findings, we hypothesized that GAD patients showed weaker connectivity between BLA and prefrontal regions, such as the medial prefrontal and anterior cingulate cortices and hyperconnectivity between the CMA and insula.

## Materials and Methods

### Participants

This study was approved by the Ethics Committee of West China Hospital, Sichuan University. A total of 38 patients with GAD and 31 age- and sex-matched HCs participated in the present study after giving their written informed consent. All participants were right-handed. GAD patients were recruited from the Mental Health Center, West China Hospital of Sichuan University. Psychiatric diagnoses were determined using the Mini International Neuropsychiatric Interview (MINI), Chinese version, by two experienced psychiatrists. Exclusion criteria were (1) age <18 years or over 65 years; (2) psychiatric comorbidity assessed using the MINI; (3) any history of cardiovascular diseases, major physical illness, or neurological disorder; (4) substance abuse or dependence; and (5) pregnancy. The Hamilton Anxiety Scale (HAMA) and the Generalized Anxiety Disorder 7-item Scale (GAD-7) were used to assess symptom severity. GAD patients and HCs also completed the State-Trait Anxiety Inventory (STAI).

### MRI Data Acquisition

MRI data were acquired via a 3-Tesla Siemens MRI system with an eight-channel phase-array head coil. Head motion was controlled using foam pads. Prior to scanning, participants were instructed to lie still with their eyes closed and not to fall asleep. The resting-state fMRI images were obtained using a gradient-echo echo-planar imaging sequence with the following parameters: repetition time = 2,000 ms, echo time = 30 ms, flip angle = 90°, slice thickness = 5 mm with no slice gap, field of view = 240 × 240 mm^2^, 30 axial slices, and 205 volumes in each run. High-resolution T1-weighted data were acquired using the following scan parameters: TR = 1,900 ms, TE = 2.28 ms, flip angle = 9°, 176 sagittal slices with slice thickness = 1.0 mm, field of view = 240 × 240 mm^2^, data matrix = 256 × 256.

### Resting-State fMRI Data Preprocessing

Resting-state data were preprocessed using the Data Processing and Analysis for Brain Imaging toolkit (DPABI, http://www.rfmri.org/dpabi). The first 10 volumes were removed to allow for signal equilibration effects. Slice timing and realignment were then performed. The Friston 24-parameter model of head motion and other sources of spurious variance (white matter and cerebrospinal fluid signals) were removed from the data to reduce the effects of non-neuronal BOLD fluctuations. Next, the images were spatially normalized to the standard Montreal Neurological Institute (MNI) space and resampled to a voxel size of 3 × 3 × 3 mm^3^. Spatial smoothing with an isotropic Gaussian kernel with a full width at half maximum of 8 mm was then applied. Subsequently, linear trend removal and temporal band-pass filtering (0.01–0.08 Hz) were performed to decrease the effects of high-frequency physiological noise and low-frequency drift. To further exclude head motion effects, scrubbing was performed with the threshold for frame-wise displacement (FD > 0.5) (26). In addition, the MRI data was ruled out in the following analysis if (1) spatial movement in any direction >2 mm or 2°, (2) mean FD >0.3 mm, and (3) the scrubbed data <5 min. According to this threshold, one GAD patient was excluded in the further analysis. Additionally, there was no significant difference in head motion (mean FD) between GAD patients and HCs.

### Amygdala Subregion Definition

The amygdala subregions of BLA and CMA in each hemisphere were obtained by calculating the maximum probability maps defined by cytoarchitecture through the SPM Anatomy Toolbox (27). The obtained BLA and CMA masks were then downsampled to 3-mm cubic voxel for functional connectivity analyses.

### Functional Connectivity Analysis

Seed-based whole-brain functional connectivity analysis was used to map the functional connectivity pattern for each amygdala subregion. First, the mean time series of each individual amygdala subregion was calculated. Then, voxel-wise correlation between each seed and each voxel of the rest of the brain were computed. Finally, the correlation coefficients were changed to z-score using Fisher's r-to-z transformation yielding individual level whole-brain FC map for each amygdala subregion.

Using SPM 12, the FC maps of each seed from the individual level analyses were then entered into a second-level group analysis that treated subjects as a random variable in a 2-by-2-by-2 full factorial analyses of variance, with subregion (BLA vs. CMA) and hemisphere (left vs. right) as within-subject factors and group (GAD vs. HC) as a between-subject factor.

As our main aim is to identify the functional connectivity difference of each amygdala subregion between GAD patients and HCs, the second stage of analysis was performed using two-sample *t*-tests with age, sex, education, and head motion as covariates. The significance threshold was set to *p* < 0.005 (uncorrected) at the voxel level and the family-wise error (FWE) correction (*p* < 0.05) at the cluster level for multiple comparisons.

To explore whether amygdala subregional FC abnormalities were associated with illness duration and symptom severity in GAD patients, we conducted partial correlation analyses between FC strength extracted from each region showing significant group differences and illness duration, HAMA, and GAD-7 (adjusted for age, sex, education, and head motion). Furthermore, associations between these FC and personality traits as assessed by STAI were examined both in the sample as a whole and separately in the GAD patients and HCs.

## Results

### Demographic and Clinical Characteristic

As shown in Table 1, GAD patients and HC groups do not differ in age and sex. As expected, the scores of the clinical rating scale identified more severe anxiety and depressive symptoms in GAD patients than in HCs. Education level in GAD patients was significantly lower than that in HCs.

**Table 1:** Subject demographics.

	**GAD**	**HC**	***P*** **-Values**
	**(***n*** = 37)**	**(***n*** = 31)**	
Age (years)	39.78 (9.38)	36.16 (9.23)	0.133
Sex (M/F)	10/27	7/24	0.673
Education (years)	11.35 (4.04)	14.00 (3.41)	0.006^*^
Duration (months)	39.65 (45.85)	–	–
HAMA	25.49 (6.51)	0.29 (0.64)	0.001^*^
HAMD	16.11 (5.75)	0.10 (0.30)	0.001^*^
GAD-7	12.26 (5.39)	1.61 (2.77)	0.001^*^
SAI	51.86 (13.52)	31.55 (8.73)	0.001^*^
TAI	53.25 (10.80)	33.00 (8.45)	0.001^*^
Mean FD	0.127 (0.060)	0.127 (0.060)	0.996

*FD, frame-wise displacement; F, female; M, male; GAD-7, Generalized Anxiety Disorder (7-item); HAMA, Hamilton Anxiety Scale; HAMD, Hamilton Depressive Scale; SAI, State Anxiety Inventory; TAI, Trait Anxiety Inventory*.

* *Represents significant differences*.

### Amygdala Subregional Functional Connectivity

**Figure 1:**
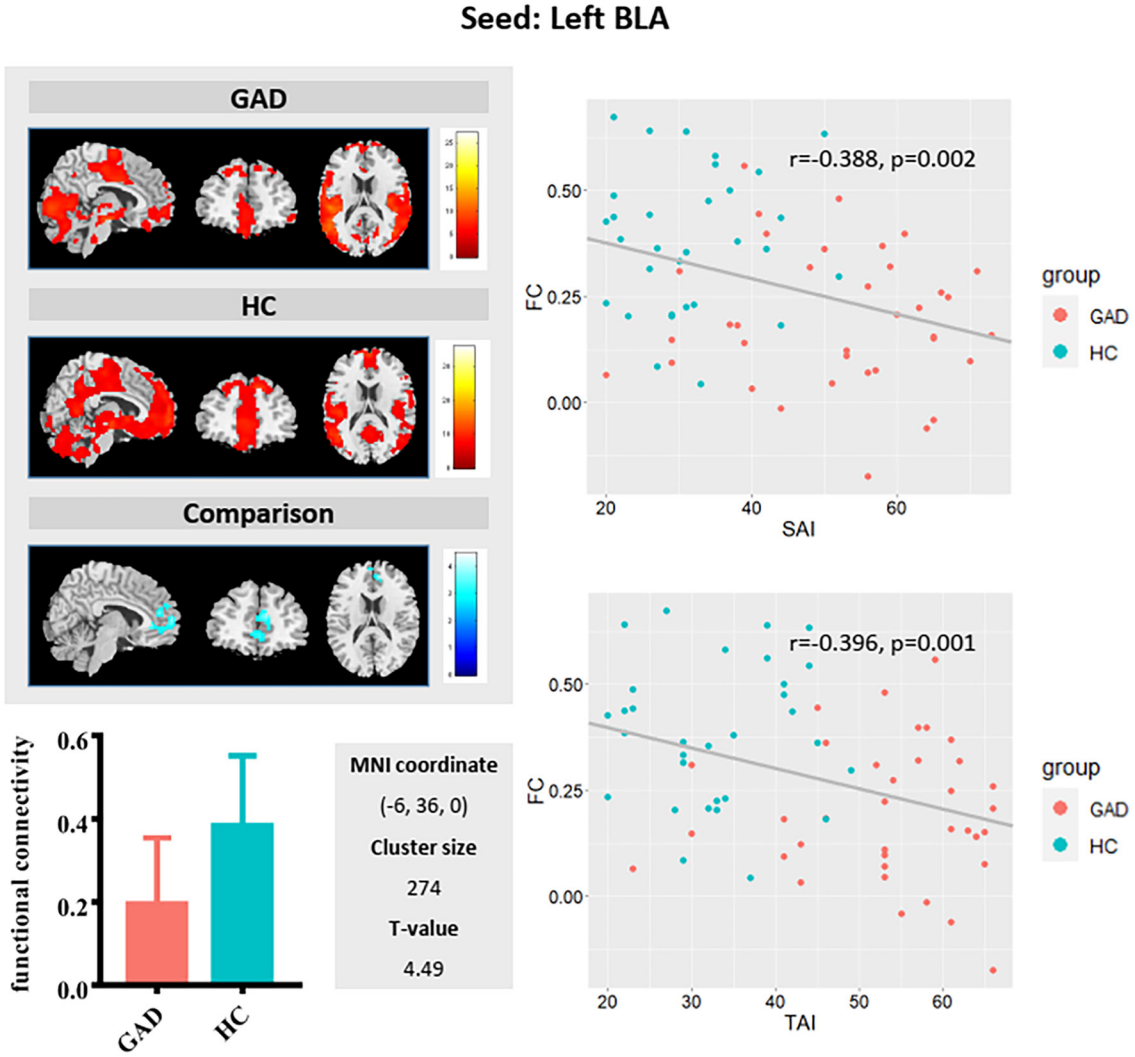
Left basolateral amygdala (BLA) functional connectivity (upper left panel) in generalized anxiety disorder (GAD) patients (first row) and in healthy controls (HCs; second row), and group differences shown in the third row. Compared with HCs, GAD patients showed weaker functional connectivity between the left BLA and anterior cingulate/medial prefrontal cortices (see bar graph in lower left panel). As shown in the scatterplot (right panel), a weaker FC between the left BLA and anterior cingulate/medial prefrontal cortices is associated with a higher-state anxiety and trait anxiety score in the sample as a whole.

**Figure 2:**
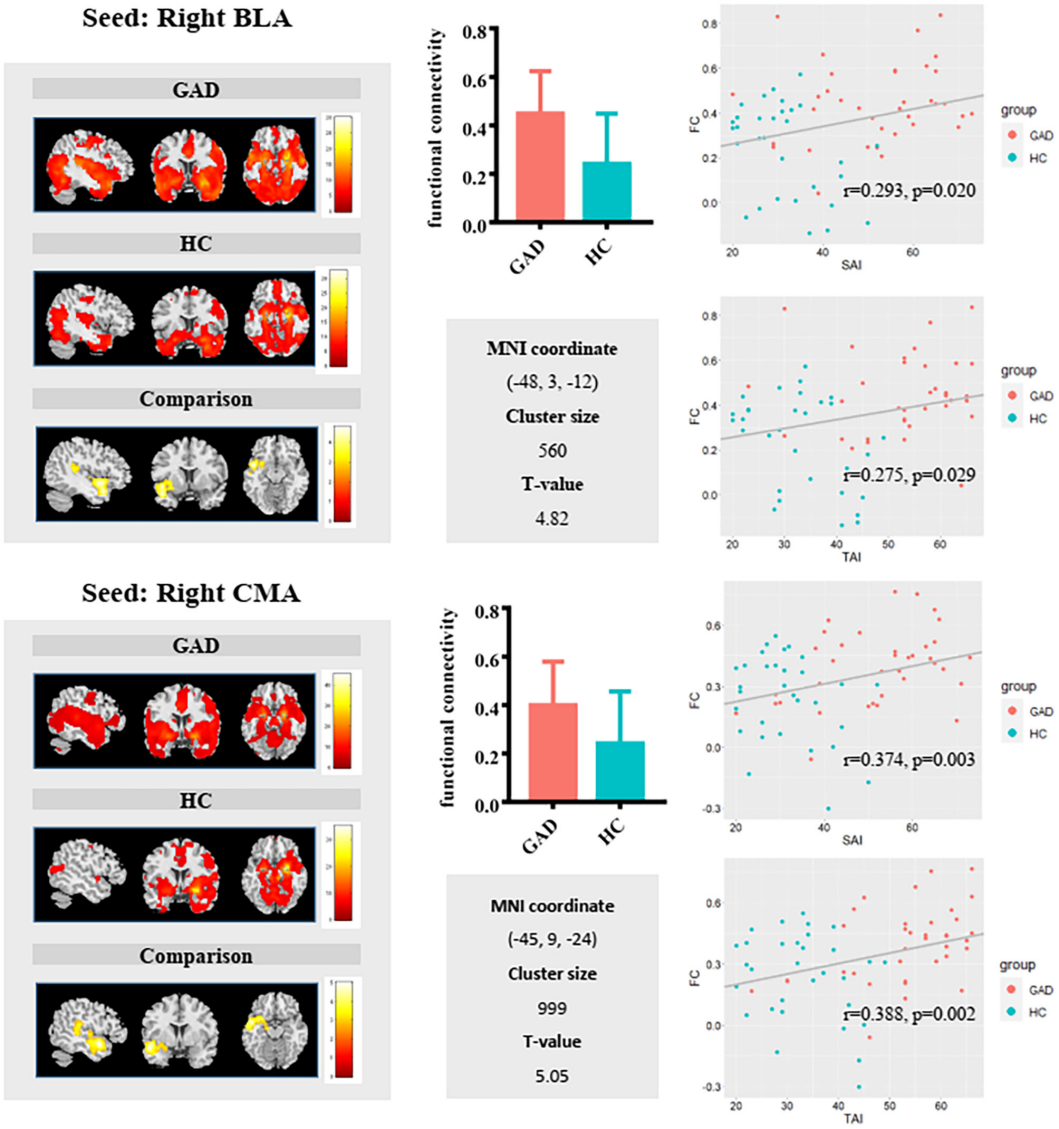
Group comparisons in FC of the right basolateral amygdala (BLA) (upper panel) and right centromedial amygdala (CMA) (lower panel) revealed relatively similar results. GAD patients exhibited significantly increased right BLA/CMA functional connectivity with a cluster including the superior temporal gyrus and insula. Additionally, the scatterplot showed positive associations between FC and State Anxiety Inventory (SAI)/Trait Anxiety Inventory (TAI) in the sample as a whole.

Full factorial analysis of variance did not identify any significant clusters. Compared with HCs, GAD patients showed weaker functional connectivity between the left BLA and left anterior cingulate/medial prefrontal cortices (Figure 1) and stronger FC between the right BLA and left superior temporal gyrus/insula, and between the right CMA and left superior temporal gyrus (Figure 2).

The FC values between the left BLA and anterior cingulate/medial prefrontal cortices correlated negatively with both state (r = −0.388, *p* = 0.002) and trait (r = −0.396, *p* = 0.001) anxiety in the whole sample group. The FC values between the right BLA and left superior temporal gyrus/insula showed positive correlation with state (r = 0.293, *p* = 0.02) and trait (r = 0.275, *p* = 0.029) anxiety. The FC values between the right CMA and left superior temporal gyrus positively correlated with state (r = 0.374, *p* = 0.003) and trait (r = 0.388, *p* = 0.002) anxiety. Additionally, positive correlations between FC of CMA with the left superior temporal gyrus/insula and trait anxiety were found in the GAD group (r = 0.355, *p* = 0.046) (Supplementary Figure 1).

## Discussion

In the current study, we revealed disrupted amygdala subregional functional connectivity in a group of drug-naive and non-comorbidity GAD patients. We found that GAD patients showed weaker functional connectivity of the left BLA with the anterior cingulate/medial prefrontal cortices and stronger FC of the right BLA and CMA with the superior temporal gyrus and insula. In addition, these functional connectivities associated with both state and trait anxiety, suggesting that amygdala subregional functional coupling may reflect the neural correlates of current fear or anxiety state and trait vulnerability for excessive fear responses.

Our finding of decreased functional connectivity between left BLA and anterior cingulate/medial prefrontal cortices in GAD supports top–down emotional dysregulation model hypothesis for the pathological mechanism of GAD (28, 29). The top–down modulation of prefrontal cortex to amygdala and related limbic structures is the neural basis of emotion regulation, and anxiety individuals experience excessive negative emotions indicating potential dysfunction of downregulation of negative emotions (28, 30). The amygdala is mainly involved in expression of emotion (31), and the prefrontal cortex is mainly associated with emotional regulation (32, 33). Besides, the anterior cingulate cortex and medial prefrontal cortex is also related to detection of emotional salience, cognition, emotion–cognition interaction, and self-consciousness and self-related mental processes (34, 35). Therefore, the amygdala–anterior cingulate cortex and amygdala–medial prefrontal cortex circuits are closely related to the occurrence and development of emotion, especially the anterior cingulate–amygdala circuit. In addition, the BLA nucleus of the amygdala–anterior cingulate cortex loop mediated associative learning process, conditioned fear, anxiety, and anticipatory anxiety (36–38). In addition, we identified negative correlation between amygdala–anterior cingulate cortex/prefrontal cortex functional connectivity and trait anxiety levels, which is consistent with previous findings that lower functional connectivity between the amygdala and anterior cingulate cortex/prefrontal cortex was associated with higher trait and state anxiety in psychiatrically healthy individuals (39–41). All the evidence indicated that the weakening of functional links between BLA and anterior cingulate cortex/medial prefrontal cortex may be the neural mechanism of disrupted top–down emotion regulation in GAD (21, 42).

The increased functional connectivity between the right BLA and CMA with the superior temporal gyrus and insula in GAD patients demonstrated that besides the high vigilance theory, GAD is also characterized by fear of threats (43, 44). A previous study found that functional connectivity between the amygdala and STG and insula was positively correlated with anxiety severity level in the GAD group (22). The superior temporal gyrus is related to the high-level cognitive process of fear experience and regulation of amygdala activity (45). Both insula and superior temporal gyrus play an important role in the nervous regulation of visceral organs to physiological conditions caused by anxiety response, such as temperature, pain, and exercise (46). Moreover, functional connectivity of the amygdala–superior temporal gyrus/insula was found to be positively correlated with trait anxiety levels suggesting that functional connectivity of the amygdala–superior temporal gyrus/insula is able to predict levels of anxiety (47–49). Therefore, we speculated that increased functional connectivity between BLA, CMA, and superior temporal gyrus/insula may be related to high worry about affairs and overreaction to sensation in GAD patients, which may be the neural basis of the abnormal physical performance of GAD patients.

Given the novelty of the current study, it also bears several limitations. First, in our study, although the non-comorbidity GAD patients were recruited to identify GAD-specific alterations, it may also lead to reducing the generalizability of our results to normal clinical setting. Second, the GAD samples are still small, and the findings need to be further validated. Finally, longitudinal studies with multimodal MRI data are warranted to better reveal the neuropathology of the GAD. Further researches are needed to clarify and extend our findings.

In conclusion, our study found disrupted left BLA–prefrontal and right BLA/CMA-temporal/insular circuits in GAD patients. More importantly, we found a negative correlation between connectivity of the amygdala–prefrontal cortex/anterior cingulate cortex circuit and positive correlation between the connectivity of the amygdala–superior temporal gyrus/insula circuit and trait anxiety levels. These findings highlight the important roles of the amygdala–frontal and amygdala–temporal circuits in the neuropathology of anxiety. This abnormal functional connectivity may underlie disrupted cognitive and affective processes in GAD patients.

## Data Availability Statement

The data that support the findings of this study are available from the corresponding author upon reasonable request.

## Ethics Statement

This study was approved by the Ethics Committee of West China Hospital, Sichuan University, and written informed consent was obtained from each participant.

## Author Contributions

MW, LC, CQ, and XH formulated the research questions. CQ and XH designed the study. HL, HX, and YM acquired the data. MW, LC, and SL analyzed the data. MW, LC, CQ, and XH wrote the article or revised it. HZ and MY reviewed the article. All authors approved the final version to be published.

## Funding

This study was supported by the National Nature Science Foundation (Grant Nos. 81671669, 81871061, and 81701328), the Science and Technology Project of Sichuan Province (Grant Nos. 2017JQ0001, 2018SZ0131, 2020YFS0231, and 2020YFS0582), the 1·3·5 Project for Disciplines of Excellence, West China Hospital, Sichuan University (Grant Nos. ZYJC21041 and ZYJC21083), a grant from the Postdoctoral Foundation of West China Hospital (Grant No. 2020HXBH041), and the Science and Technology Project of Chengdu City (Grant No. 2019-YF05-00509-SN).

## Conflict of Interest

The authors declare that the research was conducted in the absence of any commercial or financial relationships that could be construed as a potential conflict of interest.

## Publisher's Note

All claims expressed in this article are solely those of the authors and do not necessarily represent those of their affiliated organizations, or those of the publisher, the editors and the reviewers. Any product that may be evaluated in this article, or claim that may be made by its manufacturer, is not guaranteed or endorsed by the publisher.
