# Supplementary material for: Dysfunction of Resting-State Functional Connectivity of Amygdala Subregions in Drug-Naïve Patients With Generalized Anxiety Disorder
Source: Front Psychiatry. 2021 Oct 13;12:758978. doi: 10.3389/fpsyt.2021.758978 (PMC8548605; doi:10.3389/fpsyt.2021.758978)

Figure S1. A positive correlation between functional connectivity of the right centromedial amygdala (CMA) with superior temporal gyrus (STG)/insula and trait anxiety score was found in GAD patients.

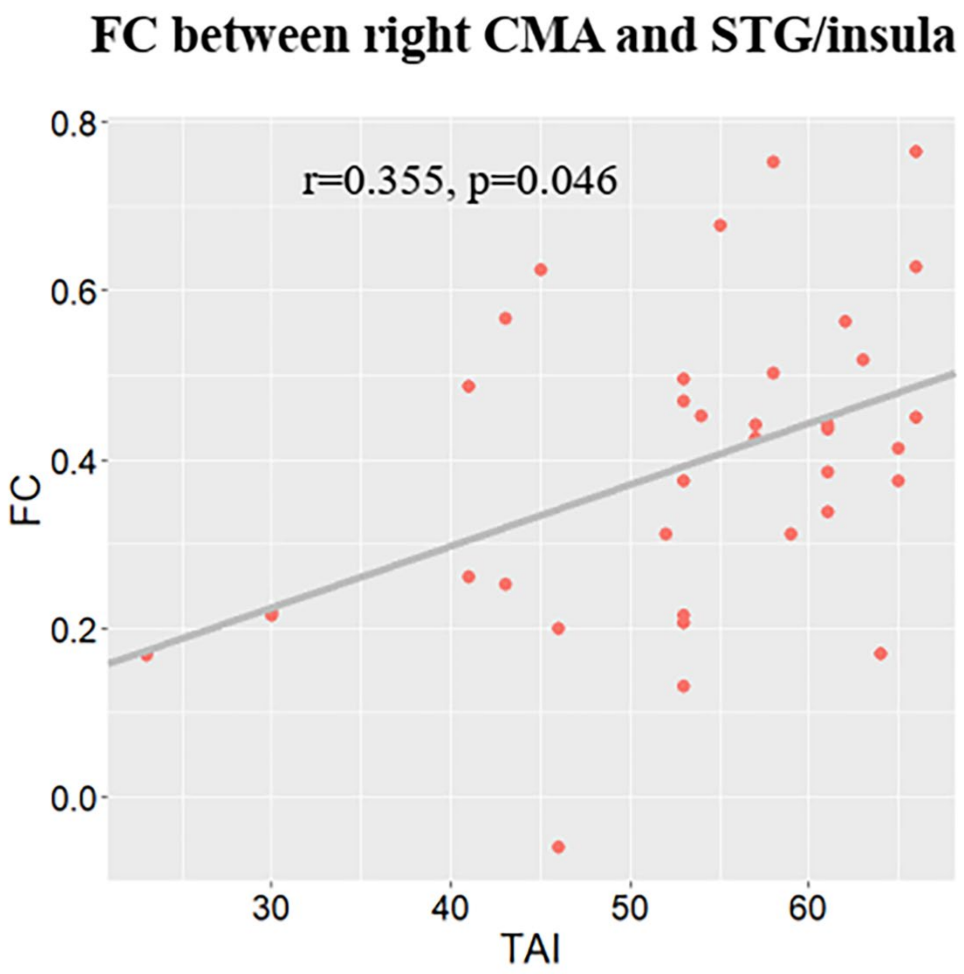

Supplement: Supplementary file 1 [file Image_1.pdf]
